# Targeted Gut Microbiota Modulation Enhances Levodopa Bioavailability and Motor Recovery in MPTP Parkinson’s Disease Models

**DOI:** 10.3390/ijms26115282

**Published:** 2025-05-30

**Authors:** Penghui Ai, Shaoqing Xu, Yuan Yuan, Ziqi Xu, Xiaoqin He, Chengjun Mo, Yi Zhang, Xiaodong Yang, Qin Xiao

**Affiliations:** 1 Department of Neurology and Institute of Neurology, Ruijin Hospital, Shanghai Jiao Tong University School of Medicine, Shanghai 200025, China; aipenghui@sjtu.edu.cn (P.A.); yybzcmu@163.com (Y.Y.); una_xu@sjtu.edu.cn (Z.X.); 18176329672@163.com (X.H.); mochengjun97@163.com (C.M.); zhangyi9102@126.com (Y.Z.); 2 Department of Geriatrics, Ruijin Hospital, Shanghai Jiao Tong University School of Medicine, Shanghai 200025, China; shaoqingfx@163.com

**Keywords:** Parkinson’s disease, gut microbiota, levodopa, fecal microbiota transplantation, pharmacomicrobiomics

## Abstract

Emerging evidence highlights the gut microbiota as a pivotal determinant of pharmacological efficacy. While *Enterococcus faecalis* (*E. faecalis*)-derived tyrosine decarboxylases (*tyrDCs*) are known to decarboxylate levodopa (L-dopa), compromising systemic bioavailability, the causal mechanisms underlying microbiota-mediated pharmacodynamic variability remain unresolved. In our study, we employed antibiotic-induced microbiota depletion and fecal microbiota transplantation (FMT) to interrogate microbiota-L-dopa interactions in MPTP-induced Parkinson’s disease (PD) mice. The study demonstrated that antibiotic-mediated microbiota depletion enhances L-dopa bioavailability and striatal dopamine (DA) level, correlating with improved motor function. To dissect clinical heterogeneity in the L-dopa response, PD patients were stratified into moderate responders and good responders following standardized L-dopa challenges. In vitro bioconversion assays revealed greater L-dopa-to-DA conversion in fecal samples from moderate responders versus good responders. FMT experiments confirmed mice receiving good-responder microbiota exhibited enhanced L-dopa bioavailability, higher striatal DA concentrations, and a heightened therapeutic effect of L-dopa relative to moderate-responder recipients. Collectively, our study provided evidence that the gut microbiota directly modulates L-dopa metabolism and microbial composition determines interindividual therapeutic heterogeneity. Targeted microbial modulation—through precision antibiotics or donor-matched FMT—is a viable strategy to optimize PD pharmacotherapy, supporting the potential for microbiota-targeted adjuvant therapies in PD management.

## 1. Introduction

Parkinson’s disease (PD), recognized as the second most common neurodegenerative disorder worldwide, is neuropathologically characterized by the progressive loss of dopaminergic neurons in the substantia nigra pars compacta (SNpc) and the presence of Lewy bodies containing aggregated α-synuclein (α-syn) [1]. The resulting dopaminergic deficit manifests clinically as cardinal motor symptoms including resting tremors, bradykinesia, and muscular rigidity [2]. Levodopa (L-dopa), the metabolic precursor of dopamine (DA), has remained the gold-standard therapy for the treatment of PD since its introduction in the 1960s [3,4]. Following intestinal absorption, L-dopa undergoes enzymatic conversion to DA via aromatic L-amino acid decarboxylase (AADC) within striatal terminals. However, its clinical utility is severely constrained by extensive peripheral metabolism. Over 95% of orally administered L-dopa is prematurely decarboxylated in the gastrointestinal tract and vasculature, resulting in low bioavailability and dose-limiting complications. Although the co-administration of peripheral AADC inhibitors (e.g., carbidopa) reduces extracerebral metabolism, approximately 50% of L-dopa still undergoes premature catabolism, highlighting unmet needs in therapeutic optimization [5,6].

Emerging evidence implicates the gut microbiota as a critical modulator of PD pathogenesis. Studies suggest that PD patients exhibit distinct microbiota compositions compared to healthy individuals [7]. Beyond disease modulation, the gut microbiota has recently emerged as a key determinant of drug pharmacokinetics through the direct enzymatic modification of xenobiotics and indirect regulation of host metabolic enzymes [8]. Notable examples include the *Eggerthella lenta (E. lenta)*-mediated inactivation of digoxin via cardiac glycoside reductases and *Faecalibacterium prausnitzii (F. prausnitzii)*-associated tacrolimus resistance in transplant recipients [9,10,11]. Moreover, research has revealed that the gut microbiota influences drug metabolism by modulating the expression and activity of metabolic enzymes in the liver and intestines [12]. Gnotobiotic studies demonstrate that microbiota depletion significantly alters hepatic cytochrome P450 (CYP3A) expression, suggesting microbiota-endobiotic crosstalk [13].

Of particular relevance to PD therapeutics, multiple bacterial species exhibit L-dopa-metabolizing capacity independent of host AADC activity. Studies discovered that tyrosine decarboxylases (*tyrDCs*), predominately found in *Enterococcous faecalis (E. faecalis)*, decarboxylated L-dopa to DA irreversibly. Subsequent bacterial metabolism by *E. lenta* further transforms dopamine into m-tyramine via dehydroxylation [5]. Moreover, *Clostridium sporogenes (C. sporogenes)* facilitates L-dopa deamination via aromatic amino acid transaminases [14]. Additionally, *Helicobacter pylori (H. pylori)* infection and small intestinal bacterial overgrowth (SIBO) impair L-dopa absorption through altered gastric motility, leading to the limitation of L-dopa efficacy [15,16,17]. While many studies have identified correlations between microbiota composition and drug metabolism or efficacy, one of the most significant hurdles in pharmacomicrobiomics is determining the causal relationships between the microbiota and drug response. By mapping individual microbiota profiles, clinicians could optimize drug therapies for PD patients, minimizing side effects and improving treatment outcomes. However, the pharmacomicrobiomic interactions in PD remain unestablished and the therapeutic potential of microbiota modulation in PD management has been underexplored.

To address these questions, we employed antibiotic-induced microbiota depletion and microbial reconstitution via fecal microbiota transplantation (FMT) in MPTP-induced PD mice. Our results demonstrated that microbiota manipulation through antibiotic treatment or FMT regulate L-dopa bioavailability and its therapeutic effects for motor symptom amelioration. These findings establish causal microbiota–drug interactions and provide preclinical rationale for developing microbiota-targeted adjuvants in PD treatment.

## 2. Results

### 2.1. Antibiotic-Induced Gut Microbiota Depletion Improved L-Dopa Bioavailability

**Figure 1:**
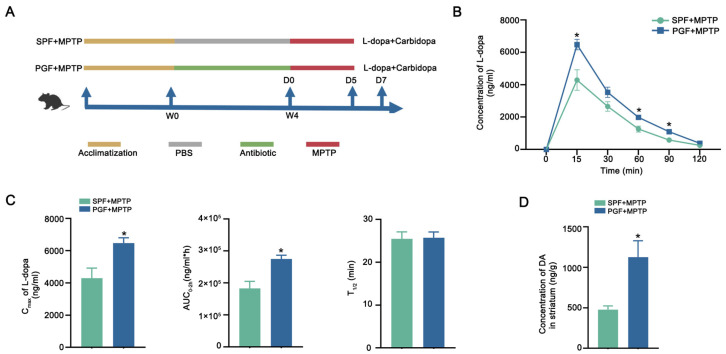
Antibiotic depletion of gut microbiota improved L-dopa bioavailability. (**A**) Schematic diagram of the experiment. (**B**) Serum concentration–time curves of serum L-dopa in each group. (**C**) C_max_, AUC_0–2h_, and T_1/2_ of serum L-dopa in each group. (**D**) Striatal DA concentration in each group. In each group, n = 5. Data are represented as means ± SEMs. *t*-test was used to determine significance. * *p* < 0.05. C_max_: maximum serum concentration. AUC_0–2h_: area under the curve from 0 to 2 h. T_1/2_: half-life. L-dopa: levodopa. DA: dopamine.

To evaluate the effect of gut microbiota on L-dopa bioavailability, the mice were divided into SPF + MPTP (n = 5) and PGF + MPTP (n = 5) groups. After a 4-week antibiotic treatment, quantitative PCR analysis demonstrated a >90% reduction in bacterial gene copies of pseudo-germ-free (PGF) mice compared to specific pathogen-free (SPF) controls (Figure S1). Following the intraperitoneal injection of MPTP, mice were orally administered a single dose of L-dopa/carbidopa (10/30 mg/kg) via gavage, with serial blood sampling conducted for pharmacokinetic profiling (Figure 1A). Notably, PGF + MPTP mice exhibited higher serum L-dopa concentrations at 15, 60, and 90 min timepoints versus SPF + MPTP controls. Pharmacokinetic modeling revealed significant increases in the maximum plasma concentration (C_max_) and systemic exposure (area under the curve from 0 to 2 h, AUC_0–2h_) while the elimination half-life (T1/2) remained comparable between groups (Figure 1C). Critically, striatal DA quantification showed higher striatal DA levels in PGF + MPTP mice than in SPF + MPTP counterparts (Figure 1D). These results demonstrated that the elimination of gut microbiota effectively increased the availability of L-dopa, thus increasing functional central nervous system (CNS) delivery.

### 2.2. Antibiotic-Induced Gut Microbiota Depletion Enhanced Therapeutic Effect of L-Dopa in PD Mice

**Figure 2:**
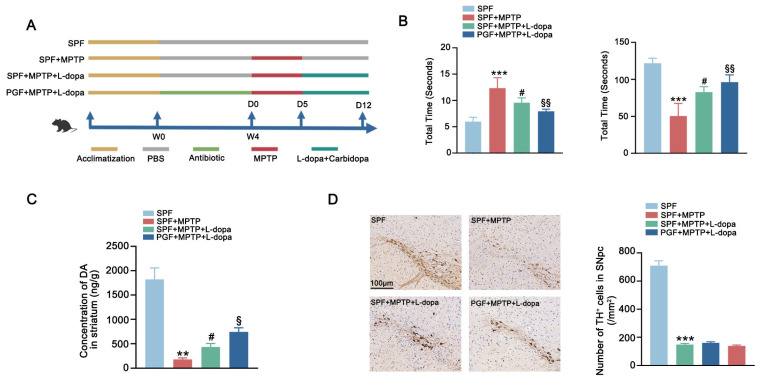
Antibiotic depletion of gut microbiota improved therapeutic effect of L-dopa in PD mice. (**A**) Schematic diagram of the experimental procedure. (**B**) Behavioral test of each group. The left figure shows the pole test and the right figure shows the rotarod test. (**C**) Striatal DA concentration in each group. (**D**) The left figure shows representative images of immunohistochemistry of TH^+^ neurons in the SNpc of mice in each group. The scale bar is 100 μm. The right figure shows the comparison of the number of TH^+^ neurons between groups. In each group, n = 5. Data are represented as means ± SEMs. The comparison between the two groups was conducted through *t*-test. The comparisons among multiple groups were performed through one-way analysis of variance followed by Tukey’s post hoc tests. * means versus SPF group, # means versus SPF + MPTP group, and § means versus SPF + MPTP + L-dopa group. ** *p* < 0.01, *** *p* < 0.001, # *p* < 0.05, § *p* < 0.05, and §§ *p* < 0.01. DA: dopamine. TH^+^: tyrosine-hydroxylase-positive. SNpc: substantia nigra pars compacta.

To evaluate the therapeutic impact of microbiota-L-dopa interactions, we implemented a 7-day therapeutic regimen (L-dopa/carbidopa) in MPTP-induced PD mice (Figure 2A). Behavioral tests revealed that SPF + MPTP mice showed a prolonged pole descent latency and reduced rotarod retention time compared with the control group. L-dopa and carbidopa treatment ameliorated the MPTP-induced impairment of motor function while the depletion of gut microbiota further enhanced the motor function recovery in the PGF + MPTP + L-dopa group compared with the SPF + MPTP + L-dopa group (Figure 2B). The level of striatal DA was significantly elevated in the SPF + MPTP + L-dopa group compared with untreated PD mice (SPF + MPTP group) whereas PGF + MPTP + L-dopa cohorts showed a greater increase compared to the SPF + MPTP + L-dopa group (Figure 2C). Further analysis of dopaminergic neurons in the SNpc showed a significant reduction in the MPTP-treated group compared to the SPF group. However, neither L-dopa therapy nor L-dopa therapy following microbiota depletion resulted in the recovery of dopaminergic neuron levels, indicating that motor improvements were driven by enhanced L-dopa pharmacodynamics rather than neuronal rescue (Figure 2D). In conclusion, removing gut microbiota can enhance the therapeutic effects of L-dopa by increasing its bioavailability.

### 2.3. FMT from Good Responders Enhanced L-Dopa Bioavailability and Therapeutic Effect

**Figure 3:**
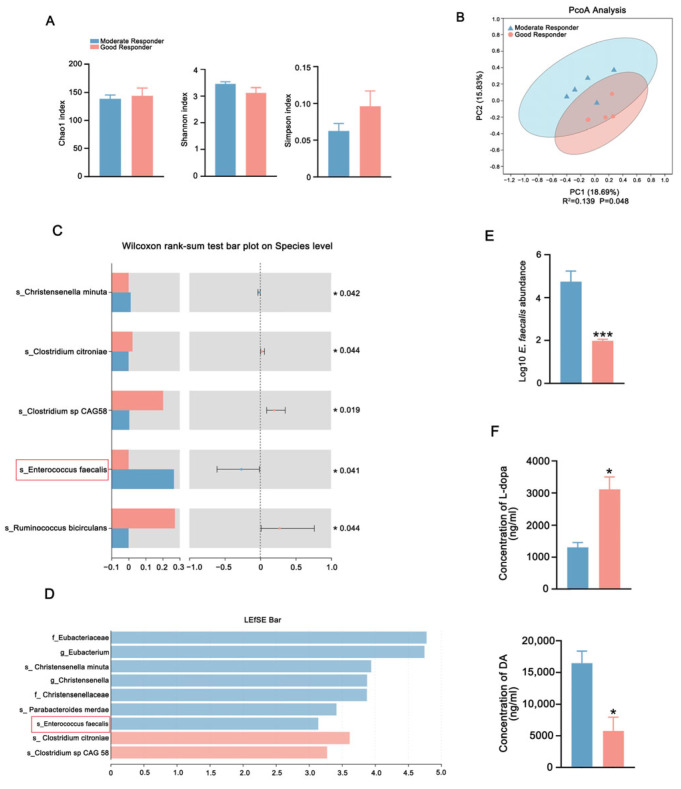
Comparison of gut microbial characteristics between the moderate responders and good responders. (**A**) Analysis of alpha-diversity in fecal microbiota of good responders and moderate responders by Chao1, Shannon, and Simpson estimators. (**B**) PCoA in fecal microbiota of good responders and moderate responders using weighted UniFrac metric based on relative abundance of OTUs. (**C**) The differential microbiota abundance of moderate responders and good responders at the species level. (**D**) LEfSe analysis computed for differentially abundant species in the feces of moderate responders and good responders. LDA scores > 2.0 are shown. (**E**) The abundances of *E. faecalis* in the feces of moderate responders and in good responders detected by qPCR analysis. (**F**) The L-dopa-metabolizing capacity of gut microbiota from good responders and moderate responders detected by fecal culture in vitro. In each group, n = 5. Data are presented as means ± SEMs. *t*-test was used to determine significance between two groups. * *p* < 0.05, and *** *p* < 0.001. PCoA: principal coordinate analysis. LEfSe: linear discriminant analysis effect size. LDA: linear discriminant analysis. *E. faecalis*: *Enterococcus faecalis*. L-dopa: levodopa. DA: dopamine.

Clinical observations revealed significant interindividual variability in L-dopa responsiveness among PD patients in our previous study [18]. To investigate microbiota-driven pharmacodynamic heterogeneity in PD, five moderate responders and five good responders to L-dopa were enrolled from our previously published L-dopa responsiveness cohort. There were no differences in clinical characteristics, medication dosages, and lifestyle variables (Table S1). The metagenomic sequencing of gut microbiota showed that the Chao1, Shannon, and Simpson indexes did not differ between the two groups, indicating similar fecal microbiota richness and diversity (Figure 3A). However, the principal coordinate analysis (PCoA) score plots revealed distinct clustering patterns, suggesting differences in the composition of gut microbiota between groups (Figure 3B). At the bacterial species level, the abundance of *E. faecalis* and *Christensenella minuta (C. minuta)* was significantly higher in moderate responders compared to good responders. Meanwhile, the abundance of *Ruminococcus bicirculans (R. bicirculans)* and *Clostridium citroniae (C. citroniae)* was greater in good responders (Figure 3C). Similarly, linear discriminant analysis effect size (LEfSe) revealed that *C. minuta*, *Parabacteroides merdae (P. merdae)*, and *E. faecalis* were enriched in moderate responders whereas *C. citroniae* was enriched in good responders (Figure 3D). Furthermore, quantitative real-time PCR analysis confirmed the increased abundance of *E. faecalis* in moderate responders (Figure 3E). To confirm the differential capacity metabolization of L-dopa by the feces of two groups, ex vivo fecal fermentation assays demonstrated that the microbiota of moderate responders metabolized L-dopa greatly, associated with excessive DA generation compared to good responders (Figure 3F).

**Figure 4:**
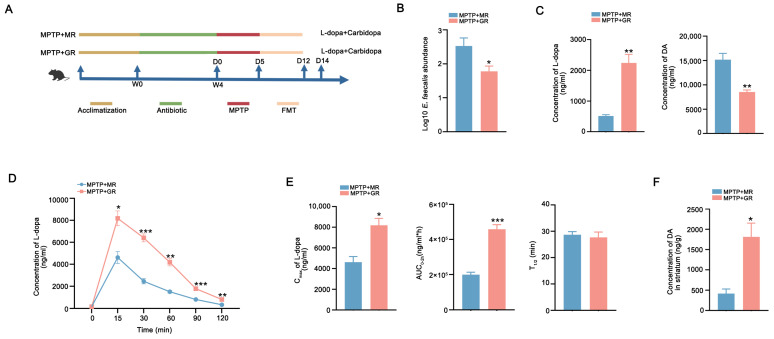
FMT from good responders improved L-dopa bioavailability in PD mice. (**A**) Schematic diagram of the experiment. (**B**) The abundances of *E. faecalis* in the feces of MPTP + MR and MPTP + GR mice detected by qPCR analysis. (**C**) The L-dopa-metabolizing capacity of gut microbiota from the MPTP + MR and MPTP + GR mice detected by fecal culture in vitro. (**D**) Serum concentration–time curves of L-dopa in two groups of mice after FMT. (**E**) The C_max_, AUC_0–2h_, and T_1/2_ of L-dopa concentration in the sera of two groups of mice after FMT. (**F**) Striatal DA concentration in two groups of mice after FMT. In each group, n = 5. Data are presented as means ± SEMs. *t*-test was used to determine significance between two groups. * *p* < 0.05, ** *p* < 0.01, and *** *p* < 0.001. L-dopa: levodopa. FMT: fecal microbiota transplantation. C_max_: maximum serum concentration. AUC_0–2h_: area under the curve from 0 to 2 h. T_1/2_: half-life. DA: dopamine.

Next, mice were colonized with donor microbiota via daily FMT for 7 days to observe the effects on the metabolism of L-dopa in vivo (Figure 4A). Notably, qPCR analysis indicated that the abundance of *E. faecalis* in the feces of mice receiving moderate-responder microbiota (MPTP + MR) was higher than in mice recipients from good-responder microbiota (MPTP + GR) (Figure 4B). Additionally, the feces of MPTP + MR mice recapitulated the metabolic signature of human donors as evidence showing enhanced L-dopa metabolism and DA production in the periphery compared to that from MPTP + good responders (Figure 4C). Conversely, mice recipients from good-responder microbiota showed revealed heightened L-dopa bioavailability with higher C_max_ and AUC_0–2h_ in the pharmacokinetic profiling in vivo (Figure 4D,E). The levels of striatal DA in recipients from good responders were higher versus moderate-responder recipients (Figure 4F). These results demonstrate that gut microbiota from good responders exhibit reduced L-dopa metabolic capacity, and consequently, FMT from these individuals enhance L-dopa bioavailability in PD mice.

**Figure 5:**
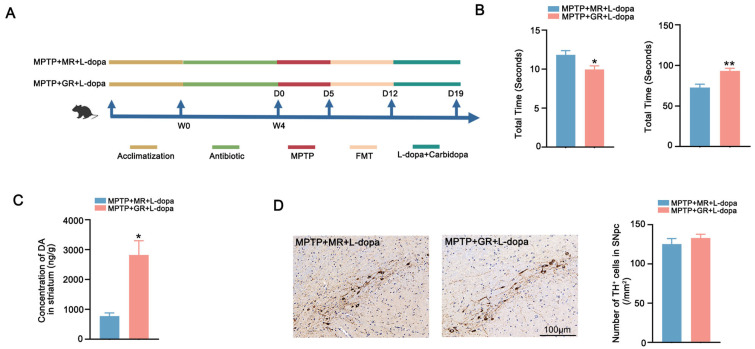
FMT from good responder enhances the therapeutic effect of L-dopa. (**A**) Schematic diagram of the experiment. (**B**) Behavioral test results of the two groups of mice after microbiota transplantation. The left figure shows the pole test and the right figure shows the rotarod test. (**C**) Striatal DA concentration in the two groups of mice after microbiota transplantation. (**D**) Representative images of immunohistochemistry of TH^+^ neurons in the SNpc of the two groups of mice after microbiota transplantation. The scale bar is 100 μm. The right figure shows the comparison of the number of TH^+^ neurons between the two groups. In each group, n = 5. Data are presented as means ± SEMs. *t*-test was used to determine significance between the two group. * *p* < 0.05; ** *p* < 0.01. DA: dopamine. TH: tyrosine hydroxylase. SNpc: substantia nigra pars compacta.

The causal links between microbial composition and L-dopa therapeutic outcomes remain to be further elucidated (Figure 5A). Behavioral assessments revealed that good responder FMT recipients (MPTP + GR + L-dopa) exhibited shorter pole descent latency and longer rotarod endurance compared to recipients of moderate responders (MPTP + MR + L-dopa) (Figure 5B). Paralleling these behavioral improvements, DA quantification demonstrated higher striatal DA concentrations in good-responder recipients (Figure 5C). The quantification of TH^+^ neuron showed no differences, confirming that the therapeutic benefits might be mediated through enhanced L-dopa bioavailability (Figure 5D). These data demonstrated that microbiota composition directly modulates L-dopa bioavailability and clinical efficacy. The transplantation of feces with a good response to L-dopa can improve the utilization and enhances the therapeutic effect of L-dopa in the PD model.

## 3. Discussion

The intricate interplay between gut microbiota and L-dopa metabolism has emerged as a pivotal factor influencing therapeutic efficacy in Parkinson’s disease [19,20]. Our findings provide compelling experimental evidence that gut microbiota directly modulates L-dopa bioavailability and the targeted manipulation of gut microbiota via antibiotic-induced depletion or that FMT significantly enhances L-dopa bioavailability and amplifies its therapeutic benefits on motor dysfunction in PD models. These results not only align with prior observations of microbial contributions to L-dopa metabolism but also introduce novel insights into microbiota-targeted interventions as a strategy to optimize PD treatment.

L-dopa absorption is restricted to the proximal small intestine (duodenum and jejunum), where it is transported into the bloodstream via a competitive transport system shared with other large neutral amino acids (LNAA) [21]. Dietary proteins, when metabolized to amino acids, competitively inhibit L-dopa absorption, highlighting the critical impact of meal composition and timing on therapeutic efficacy [22]. Additionally, peripheral enzymatic degradation by AADC and catechol-O-methyltransferase (COMT) converts L-dopa into DA and 3-O-methyldopa (3-OMD), further limiting systemic bioavailability [23]. Emerging evidence implicates specific gut microbial communities in regulating L-dopa pharmacokinetics. SIBO may influence L-dopa metabolism by increasing small intestine permeability and inflammation, potentially exacerbating motor fluctuations and unpredictable variations in PD patients [24]. These symptoms can be improved following the successful eradication of SIBO [25,26]. Similarly, *H. pylori* infection might delay gastric emptying, resulting in delayed L-dopa entry and impaired L-dopa transport in the duodenum, therefore interfering with the efficiency of L-dopa—a deficit ameliorated by pathogen clearance [15,27,28]. Our findings extend these observations by demonstrating that commensal microbiota directly metabolize L-dopa. Antibiotic-induced gut microbiota depletion significantly enhances L-dopa bioavailability with increased C_max_ and AUC_0–2h_ values of L-dopa and increases striatal DA levels, confirming microbial activity as an intestinal “first-pass” metabolic barrier. We suppose the improvement in L-dopa absorption in our study was likely driven by the suppressed activity of bacterial *tyrDCs*, notably those encoded by *E. faecalis* [5,29]. In fact, prior research has identified a compound, (S)-α-fluoromethyltyrosine (AFMT), which is recognized as a mechanism-based inhibitor of pyridoxal phosphate-dependent decarboxylases. In vitro studies have demonstrated that AFMT inhibits L-dopa decarboxylation by *TyrDC* and *E. faecalis*, as well as in complex gut microbiota samples from PD patients. In vivo studies have shown that AFMT effectively reduces the microbial metabolism of L-dopa, thereby enhancing its bioavailability in mice. These findings underscore the potential for developing L-dopa-based combination therapies targeting enzymes produced by gut microbiota to prevent microbial drug metabolism [5]. Notably, the unchanged T_1/2_ of L-dopa suggests that microbiota-mediated metabolism occurs pre-systemically in the gut rather than altering hepatic or renal clearance pathways. Critically, microbiota-depleted PD mice exhibited improved motor performance post L-dopa treatment. This benefit was directly related to elevated striatal DA levels rather than dopaminergic neuron preservation. This was because we found no differences in TH^+^ neurons in the substantia nigra between the SPF + MPTP + L-dopa and PGF + MPTP + L-dopa groups. Collectively, various bacterial pathways are involved in L-dopa metabolism, expanding the range of potential pharmacological targets to enhance L-dopa absorption. Furthermore, researchers have identified a strain of the gut Actinobacterium, *Eggerthella lenta (E. lenta)*, that is capable of selectively removing the para hydroxyl group of dopamine to yield m-tyramine, which may influence the side effects associated with peripheral L-dopa decarboxylation such as rapid hypertensive crisis. Thus, targeted gut microbiota modulation for the metabolism of L-dopa not only increases drug availability but also could reduce adverse drug responses [5,30]. Strategic microbiota modulation—via transient depletion might be a complementary strategy for suppressing microbial decarboxylation to optimize L-dopa treatment. The growing field of pharmacomicrobiomics underscores the need to characterize host–microbe–drug interactions at strain-specific resolution [8,31]. Further studies are needed to better elucidate the interaction between gut microbiota and L-dopa for individualized treatment.

Clinical experience reveals significant interindividual heterogeneity in the treatment effectiveness and medication responses of L-dopa [32,33,34]. While the disease duration, motor subtypes, and pharmacogenomic variations (e.g., COMT, dopamine receptor (DRD2), and solute carrier family 6 member 3 (SLC6A3) polymorphisms) contribute to therapeutic variability, results on the link between investigated genes and drug response phenotypes remain inconclusive, suggesting that other factors might be involved [35,36,37,38]. Multiple studies have revealed that gut microbiota interferes with pharmaceutical treatment [39,40]. Our FMT experiments have established gut microbiota as a causal association of the heterogeneity of L-dopa response for the first time. In vitro fecal cultures revealed that microbiota of moderate responders exhibited heightened L-dopa-to-DA conversion, mirroring excessive peripheral decarboxylation linked to reduced central drug bioavailability. Conversely, transplanting good-responder microbiota into PGF mice recapitulated the donors’ metabolic profile, as evidenced by the reduced degradation of L-dopa in vitro culture by microbiota from the recipient mice. Mice receiving the gut microbiota from good responders achieved enhanced L-dopa bioavailability, higher striatal DA levels, and superior motor outcomes compared to moderate-responder FMT recipients. The FMT experiment revealed that the composition of the donor’s gut microbiota influences the metabolic capacity and therapeutic efficacy of L-dopa in the recipient, underscoring the causal role of microbial composition in pharmacodynamic variability.

The beneficial effects of FMT have been clearly shown in several PD animal models, showing improvements in motor behavior, pathology, and gut microbiota features [41,42]. Currently, two preliminary studies observed improved both motor and non-motor symptoms, especially constipation, following FMT being administered [43,44]. The effectiveness of FMT on PD symptoms may be attributed to the alteration of gut microbiota and functional pathways [45,46]. In our study, we found that FMT treatment enhances the efficacy of PD medication, leading to improvements in PD symptoms. Donors with good L-dopa responsiveness exhibited gut microbiota with the reduced metabolism of L-dopa, which facilitates its absorption. Mechanically, FMT may restore gut microbiota balance and maintain intestinal homeostasis, thereby potentially enhancing drug transport efficiency [47].

Our findings position gut microbiota modulation as a tractable strategy to enhance L-dopa pharmacokinetics and clinical outcomes in Parkinson’s disease. The development of microbiota-guided interventions—such as targeted microbiota depletion or donor-selected FMT—could address the persistent challenge of therapeutic heterogeneity. However, limitations remain in our study. First, broad-spectrum microbiota depletion risks long-term dysbiosis and immune dysregulation, necessitating precision and targeted approaches. Additionally, while FMT from good responders enhanced drug bioavailability in our models, interindividual variability in microbial engraftment and functional durability requires rigorous characterization. Thirdly, establishing whether specific microbial species are directly influencing drug outcomes or whether these associations are incidental remains a key research challenge. Lastly, our results were obtained from a relatively limited sample size, and future studies will require larger samples to further validate and confirm these findings.

## 4. Materials and Methods

### 4.1. Study Approvals

This study was approved by the Research Ethics Committee of Ruijin Hospital, Shanghai Jiao Tong University School of Medicine. All participants included in this study were informed of the study’s purpose. All participants were provided informed consent forms and signed them.

### 4.2. Mouse Treatment

All animal procedures were approved by the Animal Ethics Committee of Ruijin Hospital, Shanghai Jiao Tong University School of Medicine and conducted in accordance with NIH guidelines. Mice were maintained under specific pathogen-free (SPF) conditions with suitable temperature and 12h light/dark cycles.

Mouse Experiment 1: Ten male C57BL/6 mice were randomly divided into two groups: SPF + MPTP (n = 5) group was housed in an SPF-level environment and was treated with MPTP by intraperitoneal for 5 days to induce Parkinsonism. PGF + MPTP (n = 5) group received a quadruple-antibiotic cock-tail (1 g/L ampicillin, 1 g/L neomycin, 1 g/L metronidazole, 0.5 g/L vancomycin) in drinking water for 14 days, which was confirmed as >90% microbiota depletion by weekly fecal qPCR and then followed by MPTP treatment for 5 days. On day 7, L-dopa/carbidopa (10/30 mg/kg) was orally gavaged, followed by serial blood sampling (0–120 min) from the posterior orbital venous plexus. Serum separation involved 1 h clotting at 25 °C, centrifugation (3000× *g*, 15 min, 4 °C), and storage at −80 °C for pharmacokinetic analysis. Experimental timeline and design are detailed in Figure 1A.

Mouse Experiment 2: Twenty male C57BL/6 mice were randomly assigned to four groups following acclimatization: SPF (n = 5), SPF + MPTP (n = 5), SPF + MPTP + L-dopa (n = 5), and PGF + MPTP + L-dopa (n = 5). Mice in the SPF group were housed in an SPF-level environment and orally administered sterile PBS throughout the experimental period. The SPF + MPTP group received oral administration of sterile PBS from week 1 to week 4, followed by MPTP treatment for 5 consecutive days and subsequent oral administration of sterile PBS for 1 week. The SPF + MPTP + L-dopa group received sterile PBS from week 1 to week 4, followed by MPTP treatment for 5 days and subsequent daily gavage administration of L-dopa/carbidopa (10/30 mg/kg) for 1 week. The PGF + MPTP + L-dopa group underwent a 4-week antibiotic regimen (composition as described in Experiment 1), with weekly fecal qPCR monitoring, followed by MPTP treatment for 5 days. After PD model validation, this group was administered L-dopa/carbidopa (10/30 mg/kg) daily via gavage for 7 days. Following gavage treatment, all groups underwent behavioral tests, including pole climbing and rotarod tests, to evaluate motor function. The experimental design is schematically illustrated in Figure 2A.

Mouse Experiment 3: A total of 10 matched patients were stratified into moderate responders (<40% UPDRS-III improvement post-dose) and good responders (≥40% UPDRS-III improvement post-dose) according to clinical L-dopa responsiveness detected by L-dopa challenge experiment from our previously published L-dopa responsiveness cohort. All participants were strictly matched for potential confounding factors that could potentially influence L-dopa response, including clinical characteristics, medication dosages, and lifestyle variables. Ten male C57BL/6 mice were randomly divided into MPTP + MR (n = 5) and MPTP + GR (n = 5) groups and established using fecal donors from moderate responders and good responders. Following 28-day antibiotic-induced PGF status, all mice received MPTP neurotoxin (30 mg/kg/day ×5, i.p.). PD model validation preceded daily FMT administration (200 μL/dose) for 7 days using donor stools from moderate- or good-L-dopa responders. Post FMT, mice received oral L-dopa/carbidopa (10/30 mg/kg) with serial retro-orbital blood sampling (50 μL/timepoint) at 0–120 min intervals. Serum processing involved 30 min clotting at 25 °C, centrifugation (3000× *g*, 15 min, 4 °C), and storage at −80 °C for pharmacokinetic profiling. Experimental workflow is detailed in Figure 4A.

Mouse Experiment 4: Ten male C57BL/6 mice were randomly divided into MPTP + MR + L-dopa (n = 5) and MPTP + GR + L-dopa (n = 5) groups. Following 28-day antibiotic-induced PGF status and MPTP neurotoxin administration (30 mg/kg/day ×5, i.p.), mice received donor-matched FMT via oral gavage (200 μL/day ×7) using fecal samples from PD patients stratified by L-dopa responsiveness. All the group were administered L-dopa/carbidopa (10/30 mg/kg/day ×7) after FMT with motor function assessed 24 h post-final dose via pole test and rotarod test. The experimental schematic diagram is presented in Figure 5A.

### 4.3. Quantitative Analysis of L-Dopa and DA

(I) Standard Preparation
  1. Stock Solutions: L-dopa and DA single-component stock solutions (10 ppm) prepared in 10 mM HCl and acetonitrile, respectively.
  2. Standard Mixed External Standard Solution 1: We diluted L-dopa (1 ppm) and DA (1 ppm) with acetonitrile to prepare a mixed working solution.
  3. Standard Mixed External Standard Solution 2: We diluted L-dopa (100 ppb) and DA (100 ppb) with acetonitrile to prepare a mixed working solution.
  4. Standard Mixed Internal Standard Working Solution: We diluted L-dopa-d3 (1 ppm, MCE, America) and dopamine-d4 (1 ppm, MCE, America) with acetonitrile to prepare a mixed internal-standard working solution.
  5. Calibration Standards: We took 200 µL, 100 µL, and 50 µL of Standard Mixed External Standard Solution 1 and 200 µL and 100 µL of Standard Mixed External Standard Solution 2. We added 100 µL of the mixed internal-standard working solution to each tube, vortexed thoroughly, and analyzed using the instrument to obtain five points for the standard curve.
(II) Sample Pre-treatment
  1. Culture Supernatant Sample Treatment: We centrifuged the culture supernatant at 13,000 rpm for 15 min at 4 °C. We collected 450 µL of the supernatant, added 50 µL of the mixed internal-standard working solution, vortexed thoroughly, filtered through a 0.22 µm membrane, and analyzed using the instrument.
  2. Mouse Serum Sample Treatment: We took 10 µL of serum sample, added 20 µL of the mixed internal-standard working solution, and added 70 µL of 40% acetonitrile trifluoroacetic acid solution. We vortexed for 1 min, centrifuged at 15,000 rpm for 20 min at 4 °C, collected the supernatant, and analyzed using the instrument.
  3. Mouse Brain Tissue Sample Treatment: Weigh 0.1 g of brain tissue, add 100 µL of 10 mM HCl solution, followed by 80 µL of 40% acetonitrile trifluoroacetic acid solution, and finally add 20 µL of the mixed internal-standard working solution. We homogenized using magnetic beads for 10 min, froze at −80 °C for 30 min, then centrifuged at 15,000 rpm for 20 min at 4 °C. We collected the supernatant, filtered through a 0.22 µm membrane, and analyzed using the instrument.
(III) Ultra-High-Performance Liquid Chromatography–Tandem Mass Spectrometry (HPLC-MS/MS, Shimadzu LCMS-8050, Kyoto, Japan) Conditions
  1. Liquid Phase Conditions: Mobile phase A: 2.55 mM ammonium acetate, 0.25% formic acid in water; mobile phase B: acetonitrile. Chromatographic column: YMC TICLIC-C18 (4.6 × 100 mm, 1.9 μm). Injector temperature: 10 °C; injection volume: 10 µL; flow rate: 0.2 mL/min; gradient: started with 15% B phase.
  2. Mass Spectrometry Conditions: Ionization mode: electrospray ionization (ESI) in positive and negative ion modes; spray voltage: ±3.5 kV; nebulizer temperature: 560 °C; sheath gas pressure: 30 psi; auxiliary gas pressure: 20 psi; DL tube temperature: 350 °C; ion detection mode: multiple-reaction monitoring (MRM).

### 4.4. Behavioral Tests

#### 4.4.1. Pole Test

A custom-designed wooden pole (50 cm height × 1 cm diameter) with a spherical wooden platform (diameter: 3 cm) affixed to the apex was vertically positioned within the home cage. Mice were gently placed in an inverted position on the top ball. The descent latency taken from the top of the pole into the home cage was recorded.

#### 4.4.2. Rotarod Test

Mice were placed on the rotarod apparatus and tested at a speed of 30 rpm for a maximum duration of 120 s. The latency to fall, defined as the time when the mice first fell off the rod, was recorded.

All mice underwent a 3-day pretraining period prior to the behavioral test. Each mouse performed three trials in the testing, with a 1 h interval between trials.

### 4.5. Immunohistochemical (IHC) Staining

For immunohistochemistry analysis, deparaffinized tissue sections were subjected to antigen retrieval by boiling in 10 mM citric acid buffer (pH 6.0) for 20 min. Subsequently, the sections were treated with 3% hydrogen peroxide for 10 min at room temperature to block endogenous peroxidase activity, followed by three washes with PBS. Sections were then blocked with 5% goat serum for 1 h at room temperature and incubated overnight at 4 °C with primary antibodies: tyrosine hydroxylase (CST, 58844S, 1:200). After three additional washes with PBS, the sections were incubated with horseradish peroxidase (HRP)-conjugated secondary antibody for 1 h at room temperature. Finally, the slides were stained with 3,3′-diaminobenzidine (DAB) and cover-slipped for microscopic examination.

### 4.6. Participant Recruitment

Total of 10 PD patients were enrolled in this study in the clinic of the Department of Neurology and Institute of Neurology, Ruijin Hospital, Shanghai, China. PD was diagnosed by a movement disorder specialist on the basis of the United Kingdom Parkinson’s Disease Society Brain Bank criteria [48]. The exclusion criteria were as follows: (1) atypical or secondary Parkinsonism, Alzheimer’s disease, or other central nervous system diseases; (2) levodopa intolerance; (3) history of deep brain stimulation, ablative, or lesioning procedures; (4) history of gastrointestinal tract absorption disorders including celiac disease and lactose intolerance; (5) chronic illnesses (e.g., abnormal liver enzymes, renal dysfunction, inflammatory bowel disease, malignancy, endocrine diseases, hematological diseases, or heart failure); (6) the use of antibiotics, probiotics, or proton pump inhibitors within the past 3 months; or (7) investigator judgement that the candidate was not suited to participate in the study. All participants signed the written informed consent forms.

### 4.7. Data and Sample Collection

Age, sex, and body mass index (BMI) were recorded for participants. Clinical features were collected, including disease duration, Hoehn and Yahr stage (H-Y stage), and Movement Disorder Society—Unified Parkinson’s Disease Rating Scale (MDS-UPDRS) [49]. Levodopa-equivalent daily dosages were calculated using classical methods. Fecal samples were collected with sterile fecal collection containers, and each sample was divided into aliquots as soon as possible followed by immediate storage at –80 °C prior to the following processes.

### 4.8. L-Dopa Challenge Experiment

For the specific method of the L-dopa challenge experiment, we referred to our group’s previous study [18]. Briefly, PD patients discontinued DA receptor agonists for 36 h and all other anti-Parkinsonian medications, including L-dopa, for 12 h prior to the experiment. Motor symptoms during the off-period were assessed using Part III of the Movement Disorder Society—Unified Parkinson’s Disease Rating Scale (MDS-UPDRS). At 8:00 a.m., patients received a single dose of L-dopa equivalent to 1.5 times their usual morning dose of anti-Parkinson’s medication. In this study, patients were administered 200/50 mg Madopar (L-dopa/benserazide), and motor symptoms were evaluated every 30 min based on MDS-UPDRS Part III scores for a total duration of 5 h.

The formula for calculating L-dopa efficacy is as follows:

L-dopa efficacy (%) = [(Off-period MDS-UPDRS III score − Peak MDS-UPDRS III score)/Off-period MDS-UPDRS III score] × 100%.

### 4.9. Metagenomic Sequencing

DNA extract was fragmented to an average size of about 400 bp using Covaris M220 (Gene Company Limited, Shanghai, China) for paired-end library construction. Paired-end library was constructed using NEXTFLEX Rapid DNA-Seq (Bioo Scientific, Austin, TX, USA). Paired-end sequencing was performed on Illumina NovaSeq™ (Illumina Inc., San Diego, CA, USA) at Majorbio Bio-Pharm Technology Co., Ltd. (Shanghai, China) according to the manufacturer’s instructions (www.illumina.com).

The data were analyzed on the online platform of Majorbio Cloud Platform (www.majorbio.com). Briefly, the raw sequencing reads were trimmed of adapters, and low-quality reads (length < 50 bp, with a quality value < 20 or having N bases) were removed by fastp (https://github.com/OpenGene/fastp, accessed on 30 October 2023, version 0.20.0) [50]. Reads were aligned to the human genome by BWA (http://bio-bwa.sourceforge.net, accessed on 30 October 2023, version 0.7.17) and any hits associated with the reads and their mated reads were removed [51].

The quality-filtered data were assembled with MEGAHIT (https://github.com/voutcn/megahit, accessed on 30 October 2023, version 1.1.2) [52]. Contigs with a length ≥ 300 bp were selected as the final assembling results. Open reading frames (ORFs) from each assembled contigs were predicted using Prodigal (https://github.com/hyattpd/Prodigal, version 2.6.3) and a length ≥ 100 bp of ORFs was retrieved [53]. Sequence analysis was performed using the UPARSE software (version 11) package with the UPARSE-OTU and UPARSE-OTUref algorithms. Sequences with ≥97% similarity were assigned to the same operational taxonomic units (OTUs).

### 4.10. LEfSe

Linear discriminant analysis effect size (LEfSe) was examined to determine the significant differences in microbiota at species level. The parameters were set as default: *p* values ≤ 0.05 for significance and linear discriminant analysis (LDA) score > 3.

### 4.11. Fecal Sample DNA Extraction and Quantitative Real-Time PCR

The TIANamp Stool DNA Kit (TIANGEN Biotech Co., Ltd., Beijing, China) was employed for the extraction of fecal bacterial DNA, following the manufacturer’s protocol. Microbial-species-specific primers targeting the 16S rRNA gene of *E. faecalis* (forward: CGCTTCTTTCCTCCCGAGT; reverse: GCCATGCGGCATAAACTG) were designed using Primer Premier 5.0 software. All real-time PCR amplifications were performed in a total reaction volume of 20 μL, comprising 1 μL of template genomic DNA, 10 μL of 2× T5 Fast qPCR Mix (SYBR Green I), and 10 μM of each primer. The amplification program consisted of an initial denaturation at 95 °C for 1 min, followed by 40 cycles of denaturation at 95 °C for 15 s, annealing at 60 °C for 15 s, and extension at 72 °C for 30 s. Serial dilutions of the standard plasmid DNA were used as templates to generate the real-time PCR standard curve. All quantifications were performed in triplicate, and the mean Ct value was utilized to calculate the copy numbers of *E. faecalis*. The abundances of the *E. faecalis* were expressed as log10 copies per gram of dry weight feces.

### 4.12. Metabolic Assay of L-Dopa by Fecal Incubation In Vitro

Fecal samples from PD patients and mice were processed for metabolic analysis. Specifically, approximately 1 g of feces from each PD patient group was resuspended in 10 mL of sterile phosphate-buffered saline (PBS) while 500 mg of mouse feces was resuspended in 5 mL of sterile PBS. The fecal suspensions were vigorously vortexed for 5 min and then allowed to settle at room temperature for 10 min to separate large particles and insoluble materials. Subsequently, the suspensions were diluted 1:100 into 5 mL of MEGA medium supplemented with 1 mmol of L-dopa. The mixtures were cultured under strict anaerobic conditions at 37 °C for 48 h. After incubation, the supernatant was collected, and the reaction was quenched by adding methanol. The supernatant was then centrifuged at 13,000 rpm for 10 min at 4 °C, and the resulting supernatant was subjected to HPLC-MS/MS for the quantification of L-dopa and DA [5].

### 4.13. Fecal Microbiota Transplantation

FMT was conducted following established protocols [54]. Briefly, fresh fecal samples were collected from two groups of PD patients. The feces were resuspended in sterile PBS to achieve a concentration of 0.1 g/mL and thoroughly mixed. The suspension was centrifuged at 500× *g* for 5 min at 4 °C to remove large insoluble particles, and the supernatant was collected. An equal volume of 50% sterile glycerol was added to the supernatant, which was then thoroughly mixed and aliquoted. The aliquots were stored at −80 °C until use. For the transplantation procedure, each mouse received 200 µL of the fecal suspension via daily gavage for 7 consecutive days. Control group mice were administered an equivalent volume of PBS using the same protocol.

### 4.14. Statistical Analysis

All statistical analyses were performed using GraphPad Prism 8.0 (GraphPad, Boston, MA, USA). Continuous data are presented as means ± standard errors of the mean (SEM). Comparisons between two groups were analyzed using two-tailed unpaired *t*-tests. For comparisons involving multiple groups, one-way analysis of variance (ANOVA) was conducted, followed by Tukey’s post hoc test. Categorical variables are expressed here in frequency (percentage) form and were analyzed using chi-square tests. All statistical tests were two-tailed, and a *p*-value < 0.05 was considered statistically significant.

## 5. Conclusions

Our study established the gut microbiota as a critical modifier of L-dopa pharmacokinetics and clinical efficacy in Parkinson’s disease. Targeted microbial modulation through antibiotic depletion or precision FMT enhances L-dopa bioavailability, correlating with improved striatal DA levels and motor outcomes. These insights advocate for integrating microbiota into PD treatment to maximize therapy benefits, urging a paradigm shift toward integrated host–microbe therapeutics.

## Data Availability

The original contributions presented in the study are included in the article/Supplementary Materials; further inquiries can be directed to the corresponding authors.

## Supplementary Materials

The following supporting information can be downloaded at: https://www.mdpi.com/article/10.3390/ijms26115282/s1.
