# Supplementary material for: Targeted Gut Microbiota Modulation Enhances Levodopa Bioavailability and Motor Recovery in MPTP Parkinson’s Disease Models
Source: Int J Mol Sci. 2025 May 30;26(11):5282. doi: 10.3390/ijms26115282 (PMC12155331; doi:10.3390/ijms26115282)
Supplement: Supplementary file 1 [file ijms-26-05282-s001.zip › ijms-3596910-supplementary.pdf]

Supplementary materials

Supplementary Table

Table S1. Demographic of PD patients in the study

	Moderate responder (Moderate，n = 5)	Good responder (Good，n = 5)	P
Age (years)	68.2 ± 5.0	66.8 ± 6.7	0.513
Female (n, %)	2 (40.0)	3 (60.0)	0.527
BMI (kg/m^2)	23.5 ± 1.5	24.2 ± 3.9	0.715
UPDRS-III improvement post-dose (%)	28.9 ±2.3	53.7 ± 7.8	**< 0.001**
Disease duration (y)	7.6 ±3.7	9.0 ±3.2	0.543
Hoehn and Yahr stage	2.2 ±0.8	2.1 ±0.4	0.803
MDS-UPDRS Part I score	10.8 ±6.5	10.4 ±1.3	0.896
MDS-UPDRS Part II score	15.4 ±10.4	11.8 ±5.2	0.507
MDS-UPDRS Part III score	30.8 ±16.3	35.2 ±10.7	0.628
MDS-UPDRS Part total score	60.0 ±34.3	60.0 ±19.0	0.999
LEDD (mg)	577.5 ±287.3	582.5 ±207.2	0.976

Data are presented as mean±standard deviation or frequency (percentage). Differences between groups were assessed using the chi-square test for categorical data and Mann-Whitney U test for numerical data. BMI, body mass index. MDS-UPDRS, Movement Disorder Society Unified Parkinson’s Disease Rating Scale. LEDD, levodopa equivalent daily dosage.

Supplementary Figure

A

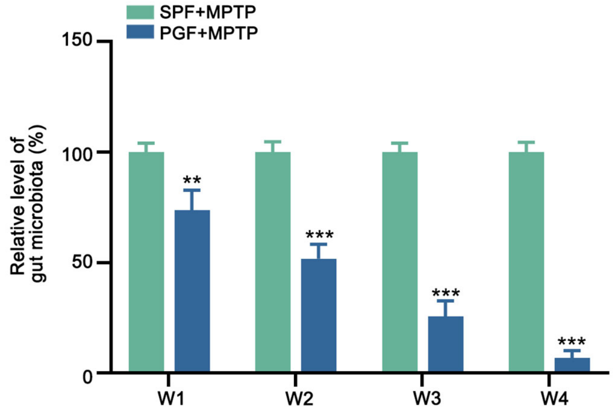

Figure S1. Relative level of gut microbiota between two group. Data are represented as the mean ± SEM. t-test was used to determine significance. **P < 0.01. ***P < 0.001. Related to Figure 1.
